# The Immunopathogenic Potential of *Arcobacter butzleri* – Lessons from a Meta-Analysis of Murine Infection Studies

**DOI:** 10.1371/journal.pone.0159685

**Published:** 2016-07-20

**Authors:** Greta Gölz, Thomas Alter, Stefan Bereswill, Markus M. Heimesaat

**Affiliations:** 1 Institute of Food Hygiene, Freie Universität Berlin, Berlin, Germany; 2 Department of Microbiology and Hygiene, Charité - University Medicine Berlin, Berlin, Germany; Fox Chase Cancer Center, UNITED STATES

## Abstract

**Background:**

Only limited information is available about the immunopathogenic properties of *Arcobacter* infection *in vivo*. Therefore, we performed a meta-analysis of published data in murine infection models to compare the pathogenic potential of *Arcobacter butzleri* with *Campylobacter jejuni* and commensal *Escherichia coli* as pathogenic and harmless reference bacteria, respectively.

**Methodology / Principal Findings:**

Gnotobiotic IL-10^-/-^ mice generated by broad-spectrum antibiotic compounds were perorally infected with *A*. *butzleri* (strains CCUG 30485 or C1), *C*. *jejuni* (strain 81-176) or a commensal intestinal *E*. *coli* strain. Either strain stably colonized the murine intestines upon infection. At day 6 postinfection (p.i.), *C*. *jejuni* infected mice only displayed severe clinical sequelae such as wasting bloody diarrhea. Gross disease was accompanied by increased numbers of colonic apoptotic cells and distinct immune cell populations including macrophages and monocytes, T and B cells as well as regulatory T cells upon pathogenic infection. Whereas *A*. *butzleri* and *E*. *coli* infected mice were clinically unaffected, respective colonic immune cell numbers increased in the former, but not in the latter, and more distinctly upon *A*. *butzleri* strain CCUG 30485 as compared to C1 strain infection. Both, *A*. *butzleri* and *C*. *jejuni* induced increased secretion of pro-inflammatory cytokines such as IFN-γ, TNF, IL-6 and MCP-1 in large, but also small intestines. Remarkably, even though viable bacteria did not translocate from the intestines to extra-intestinal compartments, systemic immune responses were induced in *C*. *jejuni*, but also *A*. *butzleri* infected mice as indicated by increased respective pro-inflammatory cytokine concentrations in serum samples at day 6 p.i.

**Conclusion / Significance:**

*A*. *butzleri* induce less distinct pro-inflammatory sequelae as compared to *C*. *jejuni*, but more pronounced local and systemic immune responses than commensal *E*. *coli* in a strain-dependent manner. Hence, data point towards that *A*. *butzleri* is more than a commensal in vertebrate hosts.

## Introduction

The gram-negative *Arcobacter* species belong to the *Campylobacteraceae* family and can be found in a plethora of habitats. In animals, *Arcobacter* spp. are mostly regarded as gastrointestinal commensals [1]. In humans, however, *Arcobacter* spp. have been shown to induce single diarrheal cases, but also disease outbreaks have been reported [2, 3]. Patients become infected by contaminated food or water and present with symptoms of acute gastroenteritis such as abdominal pain, acute or even prolonged diarrhea for up to several weeks [4, 5]. Since identification of *Arcobacter* spp. may fail in routine diagnostic laboratories, robust epidemiological data on *Arcobacter* associated human disease are lacking. In a prospective German study, for instance, no *Arcobacter* at all could be isolated in hospitalized patients suffering from community acquired acute gastroenteritis [6]. Van den Abeele and colleagues, however, reported in a large survey an *Arcobacter* prevalence of 1.3% in stool samples derived from more than 6700 Belgian enteritis patients [5]. In studies from New Zealand, Thailand and Mexico, *Arcobacter* spp. such as *A*. *butzleri* and *A*. *cryaerophilus* could be detected in 0.9–8.0% of fecal samples obtained from diarrheal patients [7–9]. Isolation rates, however, were highly depending on the respective cultivation methods applied in the respective microbiology laboratories [5]. It is therefore highly likely that the prevalence rates reported so far have been rather underestimated. In line with this, a very recent Canadian study revealed *A*. *butzleri* isolation rates of 59.6% and 0.8% from stool samples determined by PCR-based and culture-dependent methods, respectively [10]. Remarkably, neither differences could be found in fecal *A*. *butzleri* prevalences between diarrheal and non-diarrheal patients, nor did patient age, sex or place of habitation correlate with *A*. *butzleri* positive results in fecal samples derived by quantitative real-time PCR [10]. Thus, it is still an open and unanswered question whether *Arcobacter* spp. need to be regarded as ordinary commensals or rather pathogenic species. Nevertheless, based upon retrospective studies *Arcobacter* is estimated the fourth most common *Campylobacterales* genus isolated from diarrheal patients [4, 5, 11]. Furthermore, the International Commission on Microbiological Specifications for Foods have rated *A*. *butzleri* and *A*. *cryaerophilus* as serious hazards for human health among the 21 so far described *Arcobacter* species [12]. Until now information regarding the underlying mechanisms of *Arcobacter* infection and bacteria-host interactions are scarce due to lack of suitable *in vivo* infection models. Very recently our group showed in gnotobiotic (i.e. secondary abiotic) IL-10^-/-^ mice, a well-established murine model of *C*. *jejuni* infection, that *A*. *butzleri* induced intestinal and systemic immune responses [13, 14]. These immune reponses were highly dependent on Toll-like-receptor (TLR) -4 constituting the main receptor for lipooligosaccharide (LOS) and lipopolysaccharide (LPS) from gram-negative bacteria [15, 16]. In the present study we assessed the immunopathological potential of *A*. *butzeri* by comparing our published, but also so far unpublished data from gnotobiotic IL-10^-/-^ mice infected with the pathogen *C*. *jejuni* or a commensal intestinal *E*. *coli* strain [17, 18]. In this meta-analysis we aimed to unravel whether *A*. *butzleri* exhibited immunopathological features of a pathogen or a commensal.

## Materials and Methods

### Ethics statement

All animal experiments were conducted according to the European Guidelines for animal welfare (2010/63/EU) with approval of the commission for animal experiments headed by the “Landesamt für Gesundheit und Soziales” (LaGeSo, Berlin, registration numbers G0173/07, G0135/10, and G0184/12). Animal welfare was monitored twice daily by assessment of clinical conditions.

### Study design

Data were pooled from separate published [13, 14, 17, 18] as well as so far unpublished animal trials.

### Generation of gnotobiotic IL-10^-/-^ mice

IL-10^-/-^ mice (in C57BL/10 background, B10) were bred and kept in the facilities of the “Forschungseinrichtungen für Experimentelle Medizin” (FEM, Charité - Universitätsmedizin, Berlin, Germany) under specific pathogen-free (SPF) housing conditions. Gnotobiotic IL-10^-/-^ mice were generated by broad-spectrum antibiotic treatment as described earlier [19]. In brief, mice were kept in sterile cages and and had *ad libitum* access to an antibiotic cocktail consisting of ampicillin/sulbactam (1 g/L; Pfizer, Berlin, Germany), vancomycin (500 mg/L; Hexal, Holzkirchen, Germany), ciprofloxacin (200 mg/L; Hexal, Holzkirchen, Germany), imipenem (250 mg/L; Fresenius Kabi, Graz, Austria), and metronidazole (1 g/L; Braun, Melsungen, Germany) in drinking water starting at 3 weeks of age immediately after weaning and continued for 3–4 months before the infection experiment [20]. Three days prior infection, the antibiotic cocktail was replaced by sterile tap water (*ad libitum*). These so generated gnotobiotic (i.e. secondary abiotic) mice were continuously kept in a sterile environment (with autoclaved food and drinking water), handeled under strict aseptic conditions and displayed a virtually depleted gastrointestinal microbiota.

### Bacterial strains

A commensal *E*. *coli* strain was isolated from our naive and conventionally colonized C57BL/6 wildtype mice as described earlier [19]. No known virulence factors of pathogenic *E*. *coli* such as *stx-*1 and -2, *catA*, *hlyA*, *cspA*, *katP* and *astA* could be detected by PCR analysis in a reference laboratory [18]. The *A*. *butzleri* reference strain CCUG 30485 was derived from a fecal sample of a diarrheal patient [21], whereas the C1 strain was isolated from fresh chicken meat [22]. As a pathogenic reference strain, *C*. *jejuni* strain 81-176 was chosen.

### Infection of mice

Gnotobiotic IL-10^-/-^ mice were infected with 10^9^ colony forming units (CFU) of *C*. *jejuni* strain 81-176, *A*. *butzleri* reference strain CCUG 30485 or strain C1, or a commensal *E*. *coli* strain by gavage in a total volume of 0.3 mL PBS on two consecutive days (day 0 and day 1) as described earlier in more detail [13, 14, 17, 18].

*C*. *jejuni* and both *A*. *butzleri* strains were grown on Karmali-Agar and Columbia Agar supplemented wit 5% sheep blood (both from Oxoid, Wesel, Germany) for two days at 37°C under microaerobic conditions using CampyGen gas packs (Oxoid), whereas the commensal *E*. *coli* was cultivated on MacConkey agar (Oxoid) for one day at 37°C in aerobic atmosphere.

### Clinical Score

A standardized cumulative clinical score (maximum 12 points), addressing the occurrence of blood in feces (0 points: no blood; 2 points: microscopic detection of blood by the Guajac method using Haemoccult, Beckman Coulter / PCD, Krefeld, Germany; 4 points: overt blood visible), diarrhea (0: formed feces; 2: pasty feces; 4: liquid feces), and the clinical aspect (0: normal; 2: ruffled fur, less locomotion; 4: isolation, severely compromized locomotion, pre-final aspect) was used in order to assess clinical signs of infection [13, 17].

### Sampling procedures

Mice were sacrificed by isofluran treatment (Abbott, Greifswald, Germany). Then, cardiac blood was taken and tissue samples removed from spleen, liver, mesenteric lymph nodes (MLN), ileum, and colon, all under sterile conditions. Ileal and colonic *ex vivo* biopsies were collected from each mouse in parallel, for microbiological, immunohistochemical and immunological analyses. Immunohistopathological changes were determined in colonic samples that had been immediately fixed in 5% formalin and embedded in paraffin. Sections (5 μm) were stained with respective antibodies for *in situ* immunohistochemistry as described earlier [13, 20].

### Immunohistochemistry

*In situ* immunohistochemical analysis of colonic paraffin sections was performed as described previously [13, 17, 18, 23, 24]. In brief, primary antibodies against cleaved caspase-3 (Asp175, Cell Signaling, USA, 1:200), CD3 (#N1580, Dako, Denmark, dilution 1:10), FOXP3 (FJK-16s, eBioscience, 1:100), B220 (eBioscience, 1:200), and F4/80 (# 14–4801, clone BM8, eBioscience, San Diego, CA, USA, 1:50) were used [13]. For each animal, the average number of positively stained cells within at least six high power fields (HPF, 400 x magnification) were determined microscopically by a double-blinded investigator [13].

### Quantitative analysis of *bacterial* colonization and translocation

Viable *A*. *butzleri*, *C*. *jejuni* and commensal *E*. *coli* were detected in feces or at time of necropsy (day 6 p.i.) in luminal samples taken from the ileum or colon and dissolved in sterile PBS. Serial dilutions were cultured on Karmali- and Columbia-Agar supplemented with 5% sheep blood (Oxoid) for two days at 37°C under microaerobic conditions using CampyGen gas packs (Oxoid) for *A*. *butzleri* and *C*. *jejuni* detection, whereas *E*. *coli* was cultivated on MacConkey and Columbia-Agar supplemented with 5% sheep blood (Oxoid) for 48 hours in aerobic atmosphere. In order to quantitatively assess bacterial translocation, MLN, spleen, and liver *ex vivo* biopsies were homogenized in 1 mL sterile PBS, whereas cardiac blood (≈200 μL) was directly streaked onto respective solid media and cultivated accordingly. The respective weights of fecal or tissue samples were determined by the difference of the sample weights before and after asservation. The detection limit of viable pathogens by direct plating was 100 CFU per gram.

### Cytokine detection

Ileal and colonic *ex vivo* biopsies were cut longitudinally, washed in PBS, and strips of approximately 1 cm^2^ intestinal tissue placed in 24-flat-bottom well culture plates (Nunc, Wiesbaden, Germany) containing 500 μL serum-free RPMI 1640 medium (Gibco, life technologies, Paisley, UK) supplemented with penicillin (100 U/ mL) and streptomycin (100 μg/ mL; PAA Laboratories). After overnight incubation at 37°C, culture supernatants and serum samples were tested for IFN-γ, TNF, IL-6, and MCP-1 by the Mouse Inflammation Cytometric Bead Assay (CBA; BD Biosciences) on a BD FACSCanto II flow cytometer (BD Biosciences).

### Statistical analysis

Medians and levels of significance were determined using Mann-Whitney test (GraphPad Prism v6.05, La Jolla, CA, USA) as indicated. Two-sided probability (*P*) values < 0.05 were considered significant. Experiments were reproduced at least twice.

## Results

### Intestinal colonization efficacies of *E*. *coli*, *A*. *butzleri* and *C*. *jejuni* and infection-induced clinical sequelae of gnotobiotic IL-10^-/-^ mice

**Fig 1:**
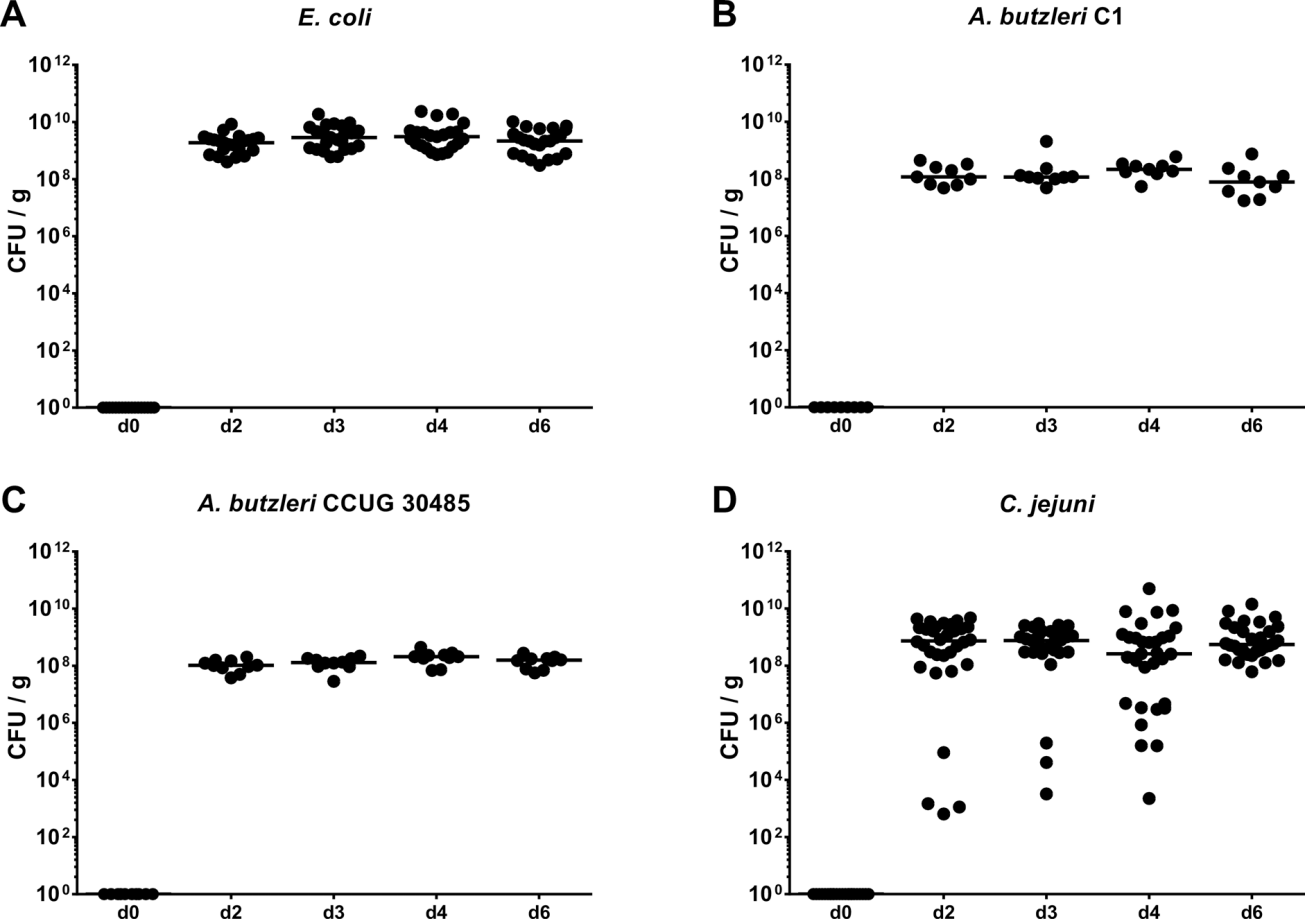
Kinetic survey of fecal bacterial shedding in perorally infected gnotobiotic IL-10^-/-^ mice. Gnotobiotic IL-10^-/-^ mice were generated by antibiotic treatment and perorally colonized either with (**A**) a commensal *E*. *coli* strain, **(B)** *A*. *butzleri* strain C1, (**C**) *A*. *butzleri* strain CCUG 30485 or **(D)** *C*. *jejuni* strain 81-176 at day 0 and day 1 by gavage. Bacterial loads were determined in fecal samples (CFU / g, colony forming units per gram) over six days (d) post infection (p.i.) by culture. Medians (black bars) are indicated.

**Fig 2:**
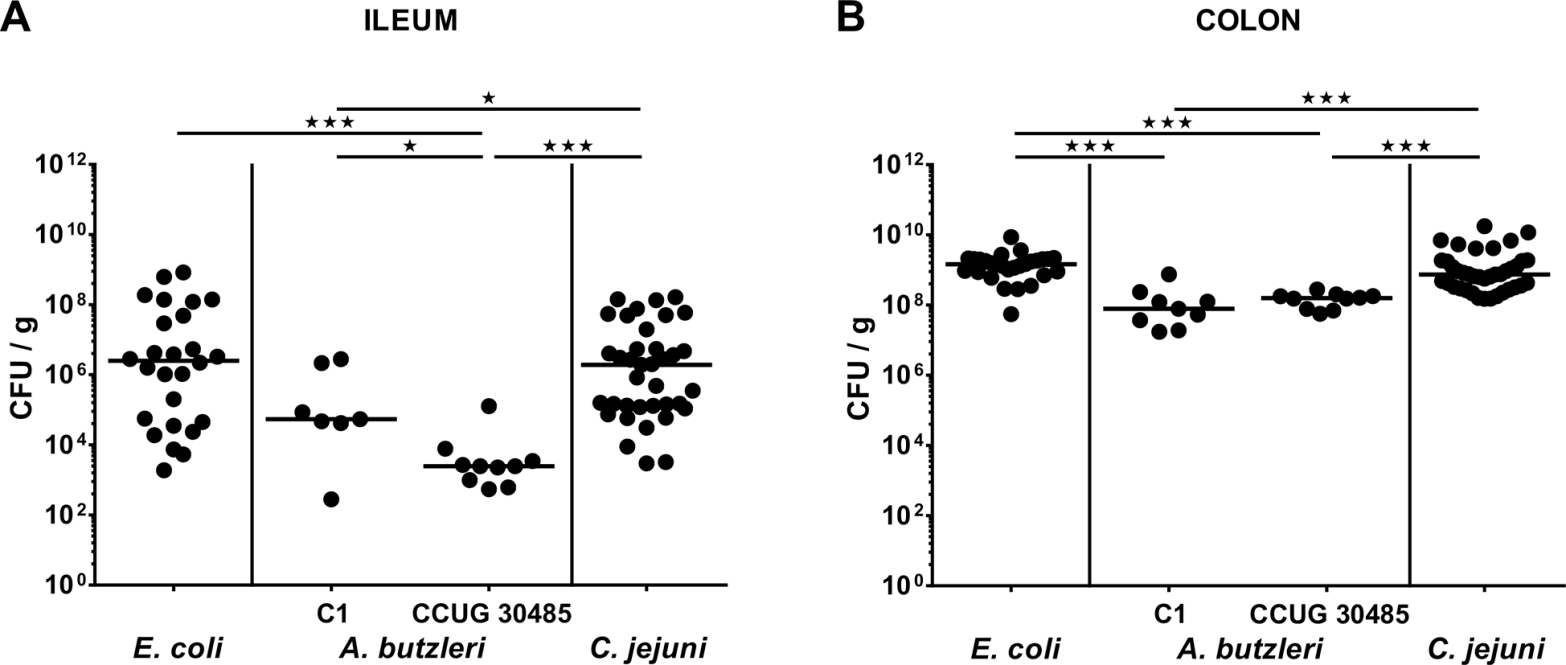
Bacterial colonization alongside the intestinal tract of perorally infected gnotobiotic IL-10^-/-^ mice. Gnotobiotic IL-10^-/-^ mice were generated by antibiotic treatment and perorally colonized either with a commensal *E*. *coli* strain, *A*. *butzleri* strain C1, *A*. *butzleri* strain CCUG 30485 or *C*. *jejuni* strain 81-176 at day 0 and day 1 by gavage. Colonization densities were determined in luminal samples derived from the (**A**) ileum and **(B)** colon (CFU / g, colony forming units per gram) at day 6 post infection by culture. Medians (black bars) and levels of significance (* p<0.05; ** p<0.01; *** p<0.001) determined by Mann-Whitney U test are indicated.

**Fig 3:**
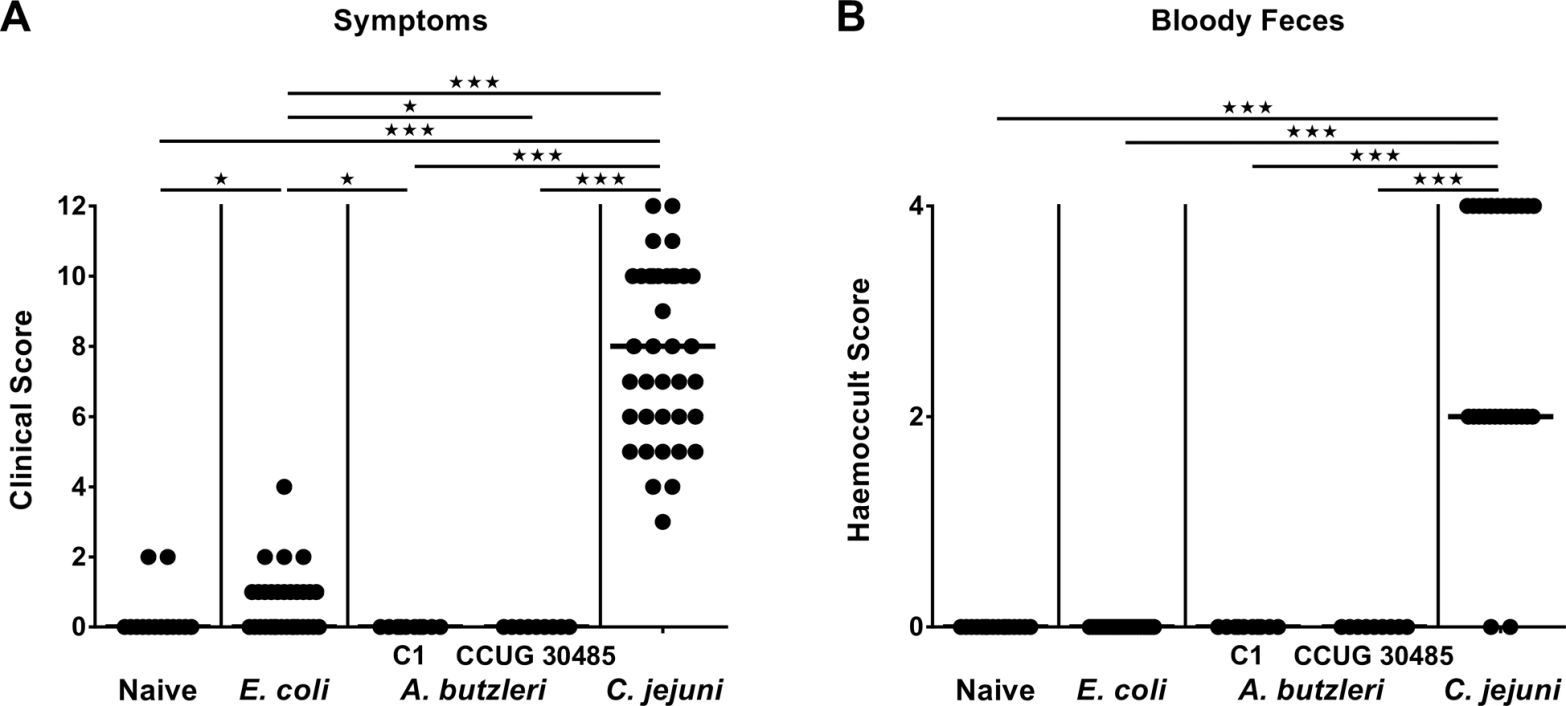
Macroscopic sequelae (disease activity) in perorally infected gnotobiotic IL-10^-/-^ mice. Gnotobiotic IL-10^-/-^ mice were generated by antibiotic treatment and perorally colonized either with a commensal *E*. *coli* strain, *A*. *butzleri* strain C1, *A*. *butzleri* strain CCUG 30485 or *C*. *jejuni* strain 81-176 at day 0 and day 1 by gavage. Naive mice served as uninfected controls. **(A)** Disease activity (symptoms) and **(B)** occurrence of blood in fecal samples (bloody diarrhea) were quantitatively assessed at day 6 postinfection applying respective standardized scores. Medians (black bars) and levels of significance (* p<0.05; *** p<0.001) determined by Mann-Whitney U test are indicated.

In the present study we aimed to compare the immunopathological potential of *A*. *butzeri* with the gram-negative intestinal pathogen *C*. *jejuni* and a commensal *E*. *coli* strain isolated from the intestinal microbiota of a conventional mouse. To address this, we applied the gnotobiotic IL-10^-/-^ mouse model generated by broad-spectrum antibiotic treatment. Following peroral infection with comparable bacterial loads of approximately 10^9^ CFU on two consecutive days (namely days 0 and 1) by gavage, *E*. *coli*, *A*. *butzleri* strains CCUG 30485 and C1 as well as *C*. *jejuni* were stably colonizing the intestinal tract of gnotobiotic IL-10^-/-^ mice, as indicated by high median bacterial loads of between 10^8^ and 10^9^ CFU per g feces (**Fig 1**). At day of necropsy (i.e. day 6 p.i.), small and large intestinal *A*. *butzleri* loads were between one and three orders of magnitude lower in colonic and ileal luminal contents, respectively, as compared to *E*. *coli* and *C*. *jejuni* infected mice (p<0.05–0.001; **Fig 2**). Analysis of the clinical outcome of infection revealed that *C*. *jejuni* infected mice were severely compromized at day 6 p.i., as indicated by increased clinical scores (**Fig 3A**), and presented with wasting ulcerative enterocolitis including bloody diarrhea (**Fig 3B**). Infection with the commensal *E*. *coli* or with either *A*. *butzleri* strain, however, induced, if any, only rather minor symptoms (**Fig 3A**), and neither gross nor occult blood could be detected in fecal samples at day 6 p.i. at all (**Fig 3B**). Taken together, following stable infection, *C*. *jejuni*, but neither *A*. *butzleri* strain nor commensal *E*. *coli* induced macroscopic disease.

### Induction of apoptosis in the colon of infected gnotobiotic IL-10^-/-^ mice

**Fig 4:**
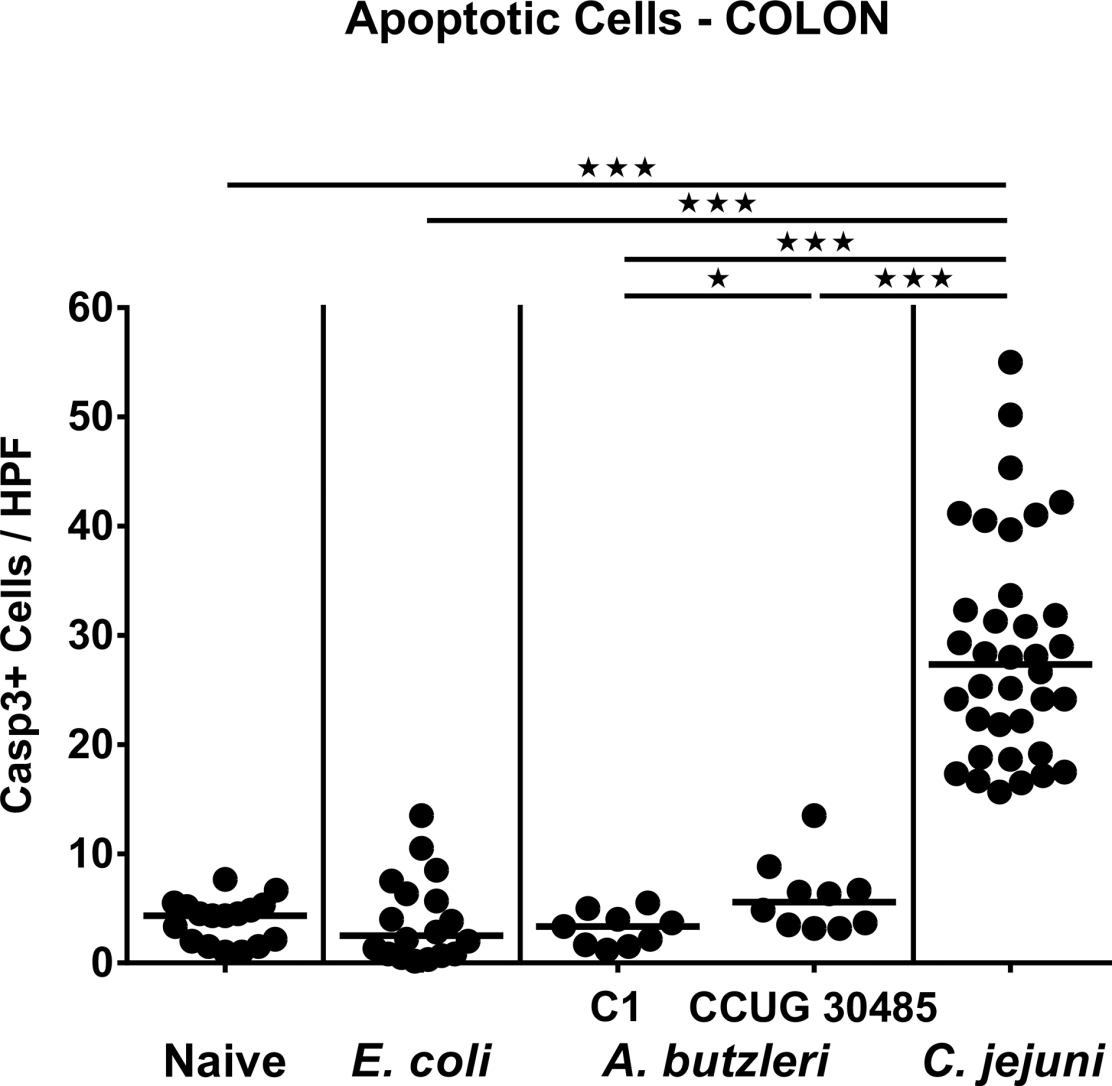
Apoptotic cells in colonic epithelium of perorally infected gnotobiotic IL-10^-/-^ mice. Gnotobiotic IL-10^-/-^ mice were generated by antibiotic treatment and perorally colonized either with a commensal *E*. *coli* strain, *A*. *butzleri* strain C1, *A*. *butzleri* strain CCUG 30485 or *C*. *jejuni* strain 81-176 at day 0 and day 1 by gavage. Naive mice served as uninfected controls. The average numbers of apoptotic cells (positive for caspase-3, Casp3) from at least six high power fields (HPF, 400x magnification) per animal were determined microscopically in immunohistochemically stained colonic paraffin sections at day 6 postinfection. Medians (black bars) and levels of significance (* p<0.05; *** p<0.001) determined by Mann-Whitney U test are indicated.

We next raised the question whether despite absence of macroscopic disease *A*. *butzleri* had the potential to induce more distinct microscopic sequelae of infection than a gram-negative commensal. Given that apoptosis is a commonly used diagnostic marker in the histopathological evaluation and grading of intestinal disease [23] and a hallmark of *C*. *jejuni* induced enterocolitis in gnotobiotic IL-10^-/-^ mice [17], we quantitatively assessed numbers of caspase-3+ cells within the colonic epithelium of infected mice. Whereas apoptotic cell numbers in the colonic mucosa increased multi-fold until day 6 following *C*. *jejuni* infection (p<0.001; **Fig 4**), there was a trend towards higher abundance of colonic apoptotic cells in *A*. *butzleri* strain CCUG 30485 as compared to *E*. *coli* infected and naive mice (n.s. after pooling of data sets; **Fig 4**). Hence, our data indicate that the potential of *A*. *butzleri* to induce macroscopic or microscopic intestinal disease does not exceed that of a commensal bacterial strain.

### Large intestinal immune cell responses in infected gnotobiotic IL-10^-/-^ mice

**Fig 5:**
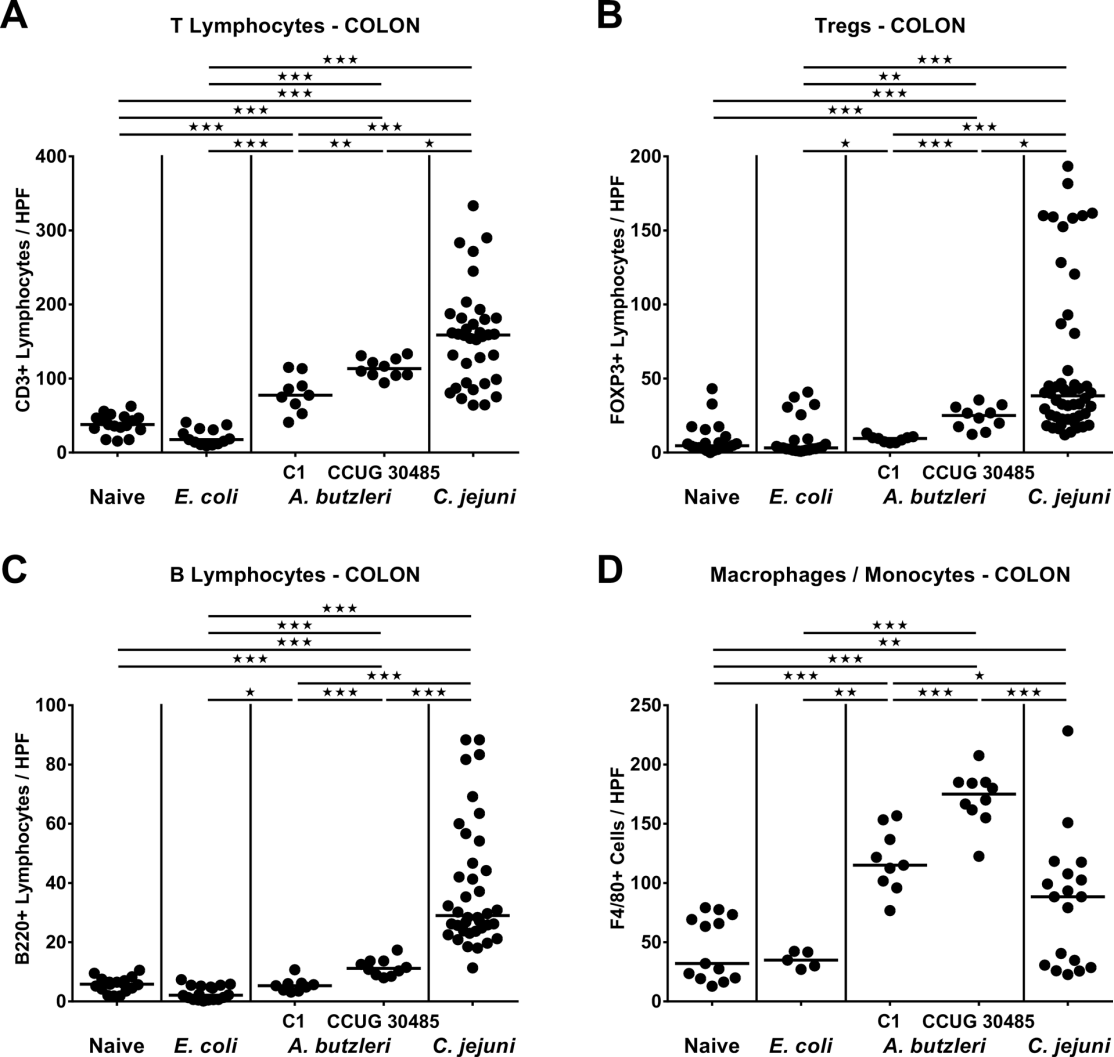
Colonic immune cell responses in perorally infected gnotobiotic IL-10^-/-^ mice. Gnotobiotic IL-10^-/-^ mice were generated by antibiotic treatment and perorally colonized either with a commensal *E*. *coli* strain, *A*. *butzleri* strain C1, *A*. *butzleri* strain CCUG 30485 or *C*. *jejuni* strain 81-176 at day 0 and day 1 by gavage. Naive mice served as uninfected controls. The average number of cells positive for **(A)** CD3 (T lymphocytes), **(B)** FOXP3 (regulatory T cells, Tregs), **(C)** B220 (B lymphocytes) and **(D)** F4/80 (macrophages and monocytes) from at least six high power fields (HPF, 400x magnification) per animal were determined microscopically in immunohistochemically stained colonic paraffin sections at day 6 postinfection. Medians (black bars) and levels of significance (* p<0.05; ** p<0.01; *** p<0.001) determined by Mann-Whitney U test are indicated.

Given that recruitment of pro-inflammatory immune cells to sites of inflammation is a key feature of infectious enteric diseases including campylobacteriosis [23], we next quantitatively assessed effector as well as innate and adaptive immune cells within the large intestinal mucosa and lamina propria by *in situ* immunohistochemical staining of colonic paraffin sections. At day 6 p.i., naive and *E*. *coli* infected gnotobiotic IL-10^-/-^ mice displayed comparable colonic numbers of CD3+ T lymphocytes, FOXP3+ regulatory T cells (Tregs), B220+ B lymphoctyes and F4/80+ macrophages and monocytes (**Fig 5**). Upon *A*. *butzleri* infection with either strain or *C*. *jejuni*, however, colonic T cell numbers increased (p<0.001; **Fig 5A**), and reached highest counts in *C*. *jejuni* infected mice. Whereas large intestinal numbers of Tregs and B lymphocytes in *E*. *coli* and *A*. *butzleri* C1 strain infected mice did not differ from naive controls, respective cell numbers increased following *A*. *butzleri* strain CCUG 30485 or *C*. *jejuni* with highest Treg and B cell counts at day 6 following *C*. *jejuni* infection (p<0.001; **Fig 5B, 5C and 5D**). Interestingly, numbers of macrophages and monocytes increased upon *C*. *jejuni* and *A*. *butzleri* infection (p<0.001; **Fig 5D**), but notably, less distinctly in *C*. *jejuni* infected mice (p<0.05–0.001; **Fig 5D**). Hence, *A*. *butzleri* (and more distinctly strain CCUG 30485 than strain C1) as well as *C*. *jejuni*, but not commensal *E*. *coli* infection, resulted in recruitment of pro-inflammatory immune cells into the colonic mucosa and lamina propria at day 6 p.i.

### Large intestinal pro-inflammatory cytokine secretion following *A*. *butzleri*, *E*. *coli* or *C*. *jejuni* infection of gnotobiotic IL-10^-/-^ mice

**Fig 6:**
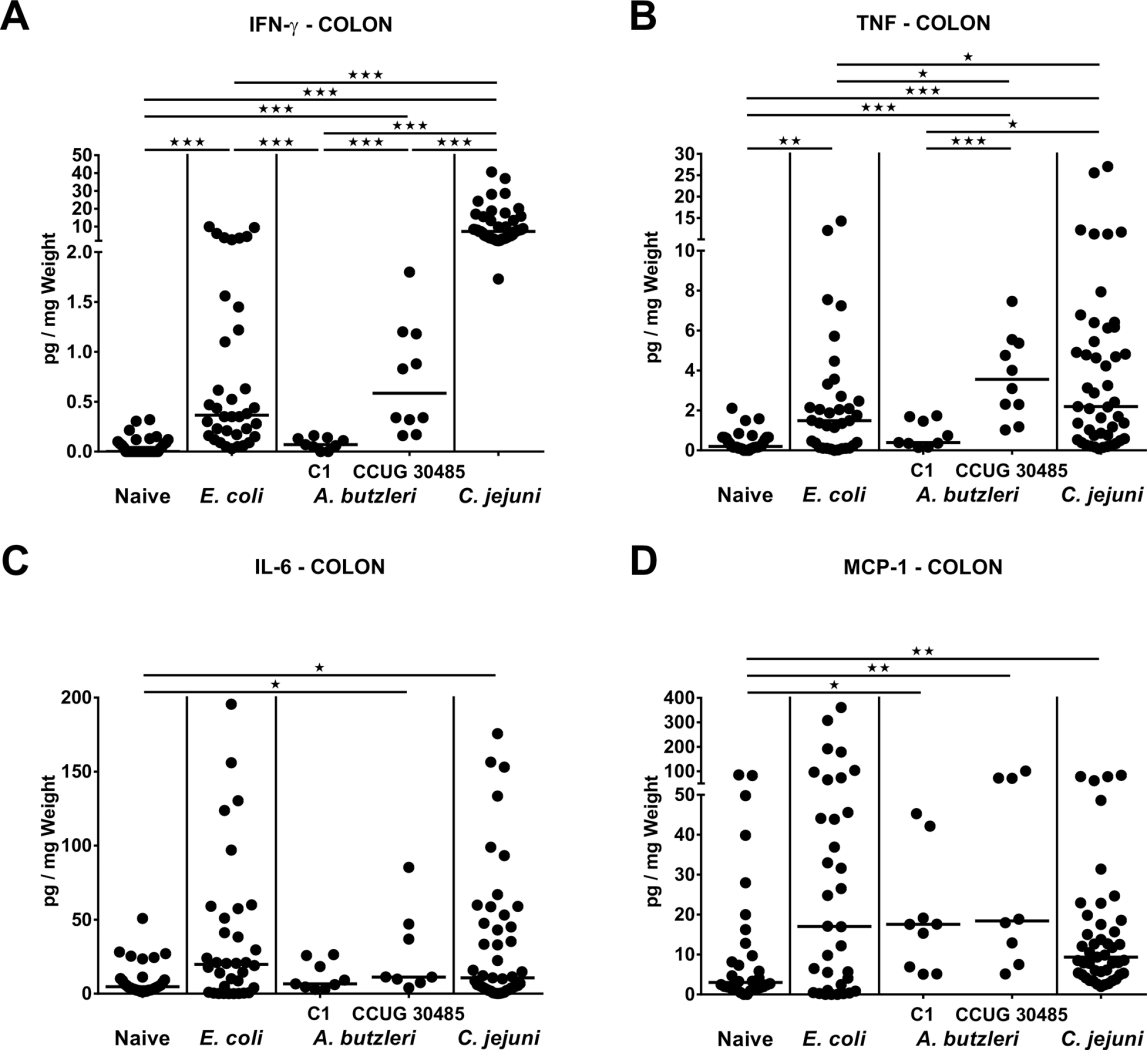
Colonic pro-inflammatory cytokine secretion in perorally infected gnotobiotic IL-10^-/-^ mice. Gnotobiotic IL-10^-/-^ mice were generated by antibiotic treatment and perorally colonized either with a commensal *E*. *coli* strain, *A*. *butzleri* strain C1, *A*. *butzleri* strain CCUG 30485 or *C*. *jejuni* strain 81-176 at day 0 and day 1 by gavage. Naive mice served as uninfected controls. Concentrations of **(A)** IFN-γ, **(B)** TNF, **(C)** IL-6, and **(D)** MCP-1 were determined in supernatants of colonic *ex vivo* biopsies at day 6 postinfection by cytometric bead assay. Medians (black bars) and levels of significance (* p<0.05; ** p<0.01; *** p<0.001) determined by Mann-Whitney U test are indicated.

We next compared colonic secretion of pro-inflammatory cytokines upon infection with either *A*. *butzleri*, *E*. *coli* or *C*. *jejuni*. Colonic IFN-γ, TNF, IL-6, and MCP-1 concentrations increased until day 6 following *C*. *jejuni* infection (p<0.05—p<0.001; **Fig 6**), whereas respective cytokines were also higher in the colon of *A*. *butzleri* strain CCUG 30485 (p<0.05–0.001; **Fig 6**) and for MCP-1 also in C1 strain infected as compared to naive mice (p<0.05; **Fig 6D**). Remarkably, colonic TNF, IL-6 and MCP-1 levels did not differ between strain CCUG 30485 and *C*. *jejuni* infected mice at day 6 p.i. (**Fig 6B, 6C and 6D**). Unexpectedly, increased large intestinal IFN-γ and TNF concentrations could also be observed in commensal *E*. *coli* as compared to uninfected mice (p<0.001 and p<0.01, respectively; **Fig 6A and 6B**), whereas only colonic TNF was lower 6 days following *E*. *coli* versus CCUG 30485 strain infection (p<0.05; **Fig 6B**). Taken together, in the large intestines distinct pro-inflammatory cytokines are increased upon *A*. *butzleri* CCUG 30485, but not C1 strain infection, and, except for INF-γ, did not differ from *C*. *jejuni* infected mice, hence supporting a strain-dependent pro-inflammatory potential of *A*. *butzleri* in the colon upon peroral infection.

### Small intestinal pro-inflammatory cytokine secretion following *A*. *butzleri*, *E*. *coli* or *C*. *jejuni* infection of gnotobiotic IL-10^-/-^ mice

**Fig 7:**
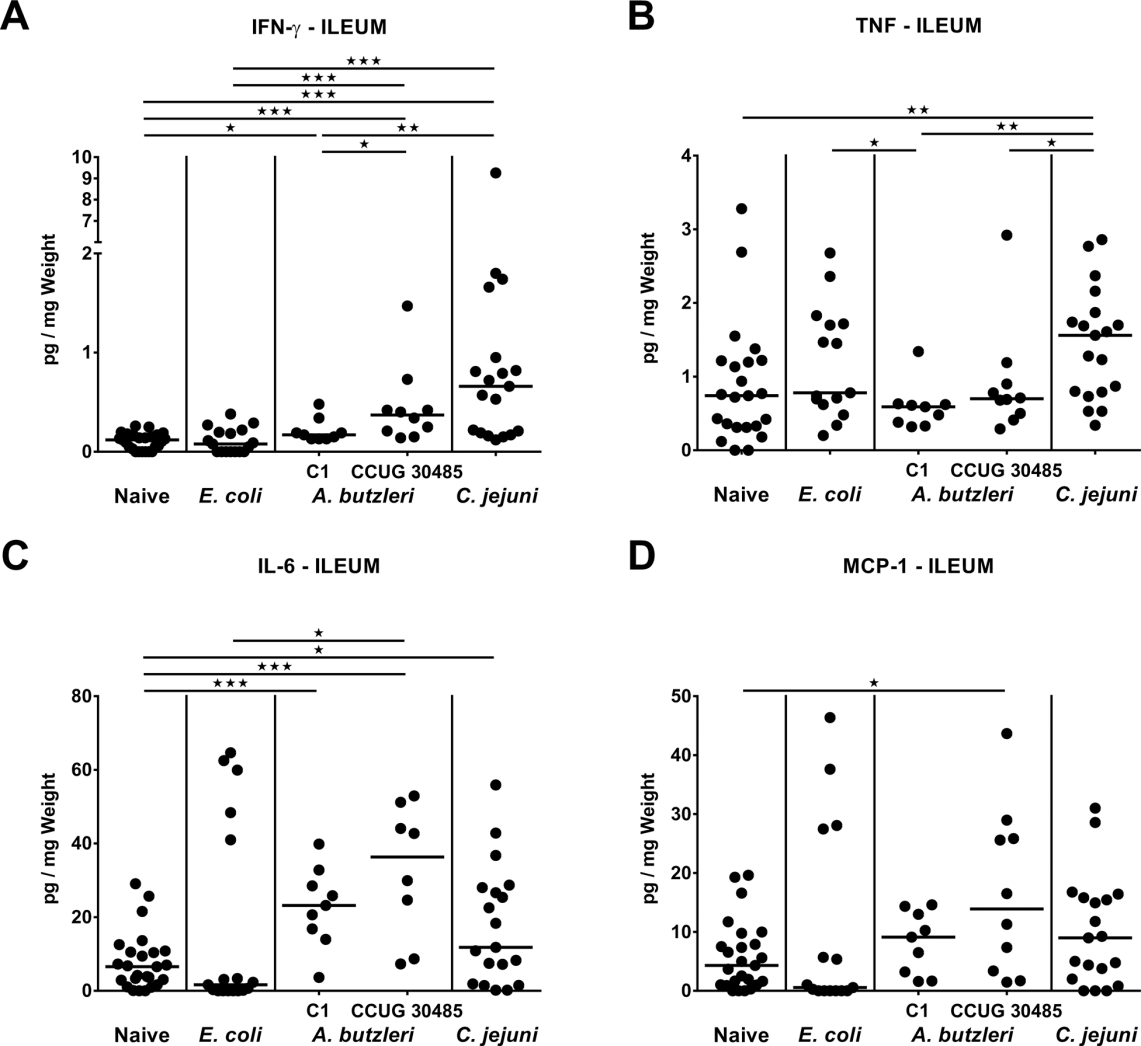
Ileal pro-inflammatory cytokine secretion in perorally infected gnotobiotic IL-10^-/-^ mice. Gnotobiotic IL-10^-/-^ mice were generated by antibiotic treatment and perorally colonized either with a commensal *E*. *coli* strain, *A*. *butzleri* strain C1, *A*. *butzleri* strain CCUG 30485 or *C*. *jejuni* strain 81-176 at day 0 and day 1 by gavage. Naive mice served as uninfected controls. Concentrations of **(A)** IFN-γ, **(B)** TNF, **(C)** IL-6, and **(D)** MCP-1 were determined in supernatants of ileal *ex vivo* biopsies at day 6 postinfection by cytometric bead assay. Medians (black bars) and levels of significance (* p<0.05; ** p<0.01; *** p<0.001) determined by Mann-Whitney U test are indicated.

Even though gnotobiotic IL-10^-/-^ mice are considered a suitable model for severe *C*. *jejuni* induced colonic disease [17, 18], we addressed whether peroral infection with the respective bacterial strains might also affect pro-inflammatory cytokine secretion in the small intestinal tract. In fact, 6 days following *C*. *jejuni* infection, increased ileal IFN-γ, TNF and IL-6, but not MCP-1 concentrations could be measured (p<0.05–0.001; **Fig 7**). Remarkably, elevated IFN-γ, IL-6 and MCP-1 levels could be determined at day 6 post CCUG 30485 strain infection (p<0.05–0.001; **Fig 7A, 7C and 7D**), that did, however, not differ from ileal secretion in *C*. *jejuni* infected mice. Moreover, both, IFN-γ and IL-6 increased upon *A*. *butzleri* C1 strain infection (p<0.05 and p<0.001, respectively; **Fig 7A and 7C**), but less distinctly for the former as compared to CCUG 30485 strain infection (p<0.05; **Fig 7A**). Taken together, *A*. *butzleri* strain C1 and more distinctly strain CCUG 30485 as well as *C*. *jejuni*, but not *E*. *coli* infection is accompanied with increased pro-inflammatory cytokines in the ileum pointing towards a pronounced, but strain-dependent pro-inflammatory potential of *Arcobacter* also in the small intestinal tract.

### Bacterial translocation to extra-intestinal and systemic compartments following *A*. *butzleri*, *E*. *coli* or *C*. *jejuni* infection of gnotobiotic IL-10^-/-^ mice

**Fig 8:**
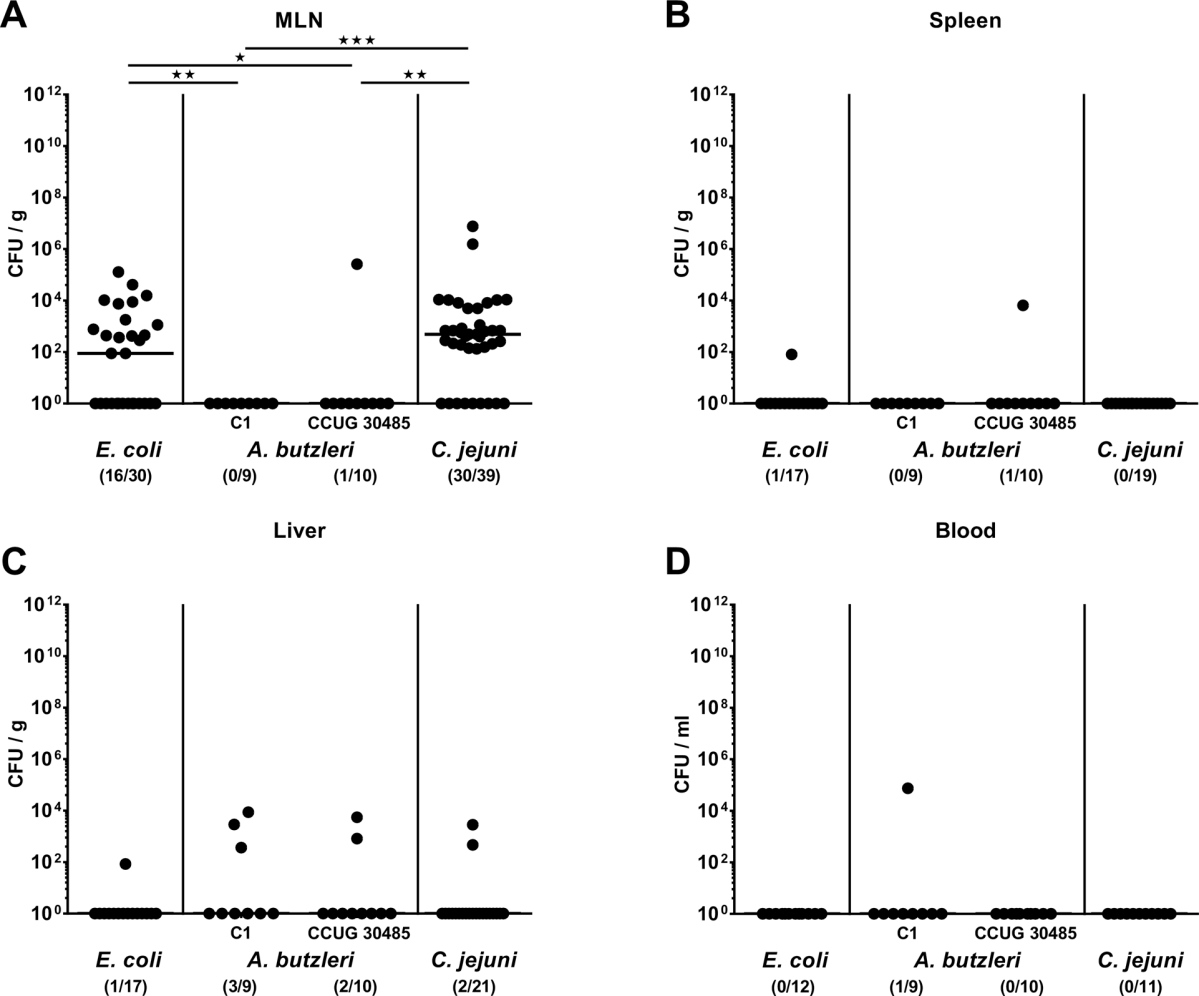
Bacterial translocation in perorally infected gnotobiotic IL-10^-/-^ mice. Gnotobiotic IL-10^-/-^ mice were generated by antibiotic treatment and perorally colonized either with a commensal *E*. *coli* strain, *A*. *butzleri* strain C1, *A*. *butzleri* strain CCUG 30485 or *C*. *jejuni* strain 81-176 at day 0 and day 1 by gavage. Naive mice served as uninfected controls. Bacterial translocation was quantitatively assessed in homogenates of **(A)** mesenteric lymph nodes (MLN) **(B)** spleen, **(C)** liver, and **(D)** cardiac blood at day 6 postinfection by culture (direct plating). Medians (black bars), numbers of mice harboring the respective bacterial species out of the total number of analyzed animals (in parenthesis), and levels of significance (* p<0.05; ** p<0.01; *** p<0.001) determined by Mann-Whitney U test are indicated.

We next addressed whether respective bacterial infections were accompanied by translocation of viable bacteria from the intestines to extra-intestinal compartments including the systemic circulation. In more than 75% of diseased *C*. *jejuni* infected mice suffering from severe enterocolitis and more than half of uncompromized *E*. *coli* infected animals, respective strains could be isolated from MLN at day 6 p.i, whereas *A*. *butzleri* was virtually undetectable (**Fig 8A**). Furthermore, 5.9% of *E*. *coli* infected, 33.3% of *A*. *butzleri* strain C1 infected, 20.0% of *A*. *butzleri* strain CCUG 30485, and 9.5% of *C*. *jejuni* infected mice harbored viable bacteria in their livers (**Fig 8C**). In systemic compartments such as the spleen and cardiac blood, however, viable bacteria were virtually undetectable (**Fig 8B and 8D**). Hence, in contrast to commensal *E*. *coli* and pathogenic *C*. *jejuni*, *A*. *butzleri* could not be cultured from MLN, whereas neither strain translocated further to extra-intestinal including systemic compartments.

### Systemic pro-inflammatory immune responses following *A*. *butzleri*, *E*. *coli* or *C*. *jejuni* infection of gnotobiotic IL-10^-/-^ mice

**Fig 9:**
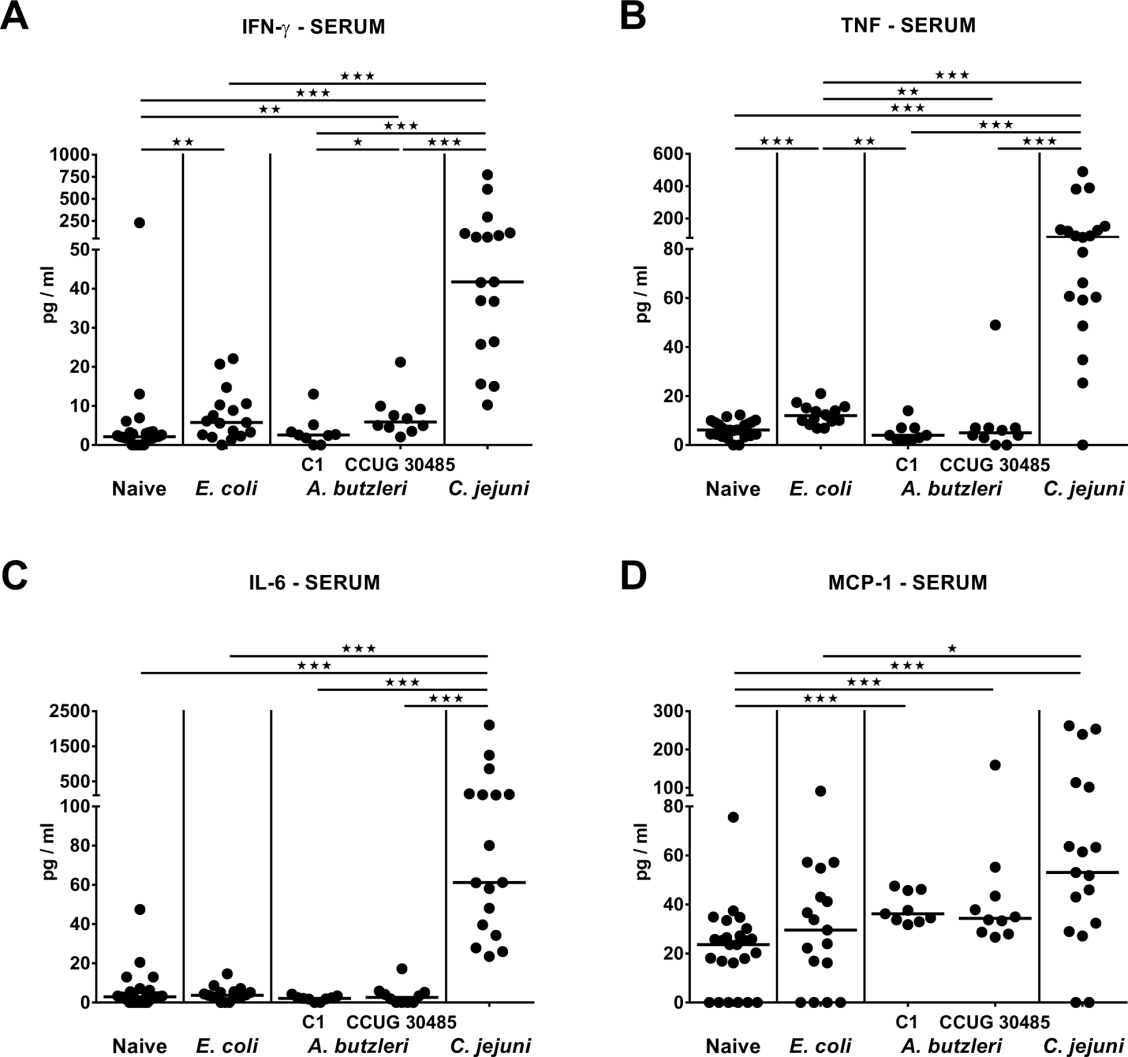
Systemic pro-inflammatory cytokine secretion in perorally infected gnotobiotic IL-10^-/-^ mice. Gnotobiotic IL-10^-/-^ mice were generated by antibiotic treatment and perorally colonized either with a commensal *E*. *coli* strain, *A*. *butzleri* strain C1, *A*. *butzleri* strain CCUG 30485 or *C*. *jejuni* strain 81-176 at day 0 and day 1 by gavage. Naive mice served as uninfected controls. Concentrations of **(A)** IFN-γ, **(B)** TNF, **(C)** IL-6, and **(D)** MCP-1 were determined in serum samples taken at day 6 postinfection. Medians (black bars) and levels of significance (* p<0.05; ** p<0.01; *** p<0.001) determined by Mann-Whitney U test are indicated.

We next addressed whether despite lack of translocating bacteria to extra-intestinal tissue sites, systemic immune responses were induced upon peroral infection. In fact, at day 6 following *C*. *jejuni* infection, increased IFN-γ, TNF, IL-6 and MCP-1 serum levels could be measured (p<0.001; **Fig 9**), whereas *E*. *coli* infection resulted in elevated IFN-γ and TNF serum concentrations (**Fig 9A and 9B**). Moreover, MCP-1 serum levels increased until day 6 following *A*. *butzleri* infection with either strain (p<0.001; **Fig 9D**) and were comparable to those obtained from *C*. *jejuni* infected mice, whereas serum IFN-γ was higher in CCUG 30485, but not C1 strain infected mice as compared to naive animals (p<0.01; **Fig 9A**). Hence, despite absence of viable bacteria from the circulation increased levels of pro-inflammatory cytokines in sera could be observed at day 6 p.i. with highest concentrations in *C*. *jejuni* infected mice, whereas increased MCP-1 serum levels were comparable in *C*. *jejuni*, *A*. *butzleri* strains CCUG 30485 and C1 infected mice.

Taken together, *A*. *butzleri* is able to induce pro-inflammatory responses in perorally infected gnotobiotic IL-10^-/-^ mice in a strain-dependent manner. Overall, however, the pro-inflammatory potential of *A*. *butzleri* is far less pronounced than for *C*. *jejuni*, but more distinct as compared to a commensal *E*. *coli* strain. Particularly in the small intestines, increased cytokine levels could be observed that did not differ between *A*. *butzleri* and *C*. *jejuni* infected mice.

## Discussion

In the present study we aimed to shed more light onto the controversy whether *Arcobacter* should be regarded as an ordinary commensal species (such as *E*. *coli)* or rather a serious intestinal pathogen (such as *Campylobacter) in vivo*. To address this, we performed a comparative survey on intestinal, extra-intestinal and systemic sequelae upon infection of gnotobiotic IL-10^-/-^ mice with a commensal *E*. *coli* strain, the intestinal pathogen *C*. *jejuni* and two different *A*. *butzleri* strains. The *C*. *jejuni* infected gnotobiotic IL-10^-/-^ mice developed wasting, non-selflimiting acute enterocolitis within one week [17, 18, 20, 24], whereas mice infected with a commensal *E*. *coli* strain did not exhibit any macroscopic or microscopic sequelae [17, 18]. As upon *E*. *coli* challenge, *A*. *butzleri* infected mice were clinically virtually uncompromized. This is rather surprising given that *in vitro* studies revealed adhesive, invasive and also cytotoxic properties of *A*. *butzleri* [22, 25–31]. Furthermore, *A*. *butzleri* infection of a human colon cell line resulted in a compromized epithelial barrier pointing towards a potential mechanism by which diarrhea is induced in *Arcobacter* infected humans [32].

Notably, before our previous reports on *A*. *butzleri* infected gnotobiotic IL-10^-/-^ mice [13–16], only one single *in vivo* study in mice had been published showing that the adherent properties of initially low-adherent *A*. *butzleri* strains were enhanced upon serial intraperitoneal passages [33]. Our murine *A*. *butzleri* infection studies, however, clearly revealed that despite absence of overt gross disease, distinct infection-induced intestinal, extra-intestinal and even systemic sequelae could be observed in an *A*. *butzleri* strain dependent manner [13–16]. These results indicate that gnotobiotic IL-10^-/-^ mice might serve as infection model to investigate *Arcobacter*-host interactions to some extent. One could argue, however, that differences in phenotypes observed in *C*. *jejuni* and *A*. *butzleri* infected mice might have been due to differences in bacterial colonization status of mice, given that *A*. *butzleri* loads in the large and small intestines were between 2 and 3 orders of magnitude lower as compared to *C*. *jejuni* (but also *E*. *coli*). Considering the high bacterial burdens of 10^8^–10^9^ CFU viable bacteria per gram luminal colon sample and 10^3^–10^6^ CFU per gram luminal ileum sample however, it is questionable whether the observed differences might have such an biological impact explaining the discrepancies in disease outcome.

Despite the lack of clinical and histopathological sequelae, however, *A*. *butzleri* induced a marked influx of effector cells as well as of innate and adaptive immune cells into the colonic mucosa and lamina propria of infected gnotobiotic IL-10^-/-^ mice, again in a strain-dependent fashion. Increases in Tregs, T and B lymphocytes were more pronounced following *C*. *jejuni* as compared to *A*. *butzleri* infection, but interestingly, the other way round was true for macrophages and monocytes. These results are well in line with leukocytic infiltrates that were observed in the intestinal lamina propria of *A*. *butzleri* infected albino rats [34]. It is tempting to speculate that these innate immune cells eradicate the bacteria and limit the systemic outcome of arcobacteriosis. This assumption is further supported by the fact that viable *A*. *butzleri* were virtually undetectable in MLN and extra-intestinal including systemic compartments.

Furthermore, increased colonic abundances of immune cells were accompanied by elevated concentrations of pro-inflammatory cytokines, not only in the large, but also small intestines following *C*. *jejuni* as well as *A*. *butzleri* infection. These findings are in line with results from an *in vitro* study demonstrating that *A*. *butzleri* infection of THP-derived macrophages resulted in an increased expression of pro-inflammatory cytokines including TNF and IL-6 [35]. Remarkably, despite the devastating and non-self-limiting phenotype following *C*. *jejuni*, but not *A*. *butzleri* infection, levels of distinct pro-inflammatory cytokines were comparable in intestinal and even in systemic compartments of *C*. *jejuni* or *A*. *butzleri* infected mice as indicated by comparable IL-6 and MCP-1 concentrations in ileum and colon, and the latter additionally in serum samples. Hence, *A*. *butzleri* induce not only intestinal, but also systemic immune responses, and this exceeds the pathogenic properties of a “merely” bacterial commensal.

Overall, the observed *A*. *butzleri* induced immune responses were more pronounced upon strain CCUG 30485 (initially isolated from a diarrheal patient) as compared to strain C1 (derived from fresh chicken meat) as indicated by higher abundances of apoptotic and immune cell populations in the colonic mucosa and higher pro-inflammatory cytokine levels such as TNF (in colon) and IFN-γ (in colon, ileum and serum) in strain CCUG 30485 versus strain C1 infected mice. It is even highly likely that different *A*. *butzleri* strains induce distinct host-dependent immune responses given that in humans some strains induce overt disease, whereas in chickens other strains behave like commensals [36]. This is supported by *in vitro* results revealing that different *A*. *butzleri* strains exerted different adhesive and invasive properties [22, 25, 31, 37], even though no direct correlation between respective phenotypes and corresponding gene patterns or functional adhesion and invasion associated gene domains could be found [22, 25]. Nevertheless, both *A*. *butzleri* strains applied in our study exerted similar virulence gene patterns and comparable capabilities of adhesion and invasion *in vitro* [22, 31].

Previous *in vivo* studies revealed that the virulence potential of *Arcobacter* was not only strain-dependent, but also correlated with host factors such as animal species and breed. For instance, certain turkey strains such as Beltsville white turkeys could be colonized by *A*. *butzleri* with variable loads and displayed mortality rates in a strain dependent manner, whereas *A*. *butzleri* was unable to readily colonize turkey poults and conventional chicken [38].

Recent investigations revealed that *A*. *butzleri* induced small and large intestinal as well as extra-intestinal and systemic immune responses were TLR-4 dependent [15, 16]. The fact that *Arcobacter* strains express variable LPS or LOS structures might further determine whether a specific strain rather acts as a pathogen or a commensal in a susceptible or resistant host. To date, however, neither *A*. *butzleri* LOS nor LPS have been isolated. In halophilic *A*. *halophilus*, however, the carbohydrate backbone of LOS has been characterized in detail [39].

**In conclusion**, *A*. *butzleri* induce less distinct pro-inflammatory sequelae as compared to *C*. *jejuni*, but more pronounced local (i.e intestinal) and systemic immune responses than commensal *E*. *coli* in a strain-dependent manner. Overall, these results are in line with the relatively low pathogenic potential of *A*. *butzleri* observed in humans, but do, in fact, point towards a immunopathogenic potential of *A*. *butzleri* in vertebrate hosts in general.
